# Group II metabotropic glutamate receptor activation attenuates acid-sensing ion channel currents in rat primary sensory neurons

**DOI:** 10.1016/j.jbc.2023.102953

**Published:** 2023-01-31

**Authors:** Qing Li, Ting-Ting Liu, Wen-Long Qiao, Jia-Wei Hao, Qing-Rui Qin, Shuang Wei, Xue-Mei Li, Chun-Yu Qiu, Wang-Ping Hu

**Affiliations:** 1 School of Pharmacy, School of Basic Medical Sciences, Xianning Medical College, Hubei University of Science and Technology, Xianning, Hubei, P R China; 2 Department of Physiology, Hubei College of Chinese Medicine, Jingzhou, Hubei, P R China

**Keywords:** group II metabotropic glutamate receptor, acid-sensing ion channel, current, primary sensory neuron, nociceptive behavior, ΔVm, membrane potential depolarization, AP, action potential, ASIC, acid-sensing ion channel, CHO, Chinese hamster ovary, DRG, dorsal root ganglion, I_pH_, proton-gated current, L-CCG-I, (2S,1′S,2′S)-2-(carboxycyclopropyl)glycine, LY, LY354740, MES, 4-morpholineethanesulfonic acid, mGluRs, metabotropic glutamate receptors, pH_0.5_, pH for half-maximal activation, PTX, pertussis toxin, TRPV1, transient receptor potential vanilloid type 1

## Abstract

Acid-sensing ion channels (ASICs) play an important role in pain associated with tissue acidification. Peripheral inhibitory group II metabotropic glutamate receptors (mGluRs) have analgesic effects in a variety of pain conditions. Whether there is a link between ASICs and mGluRs in pain processes is still unclear. Herein, we show that the group II mGluR agonist LY354740 inhibited acid-evoked ASIC currents and action potentials in rat dorsal root ganglia neurons. LY354740 reduced the maximum current response to protons, but it did not change the sensitivity of ASICs to protons. LY354740 inhibited ASIC currents by activating group II mGluRs. We found that the inhibitory effect of LY354740 was blocked by intracellular application of the G_i/o_ protein inhibitor pertussis toxin and the cAMP analogue 8-Br-cAMP and mimicked by the protein kinase A (PKA) inhibitor H-89. LY354740 also inhibited ASIC3 currents in CHO cells coexpressing mGluR2 and ASIC3 but not in cells expressing ASIC3 alone. In addition, intraplantar injection of LY354740 dose-dependently alleviated acid-induced nociceptive behavior in rats through local group II mGluRs. Together, these results suggested that activation of peripheral group II mGluRs inhibited the functional activity of ASICs through a mechanism that depended on G_i/o_ proteins and the intracellular cAMP/PKA signaling pathway in rat dorsal root ganglia neurons. We propose that peripheral group II mGluRs are an important therapeutic target for ASIC-mediated pain.

Glutamate and glutamate receptors play a key role in pain. Group II metabotropic glutamate receptors (mGluRs, including mGluR2 and mGluR3) have been found to have antinociceptive effects in preclinical models of acute and chronic pain (1). Pharmacological activation of group II mGluRs relieves nociceptive behaviors in inflammatory pain and mechanical allodynia in neuropathic pain (2, 3). Nociceptive behaviors are further exacerbated by group II mGluR antagonists in inflammatory pain conditions, indicating that endogenous mGluRs are activated (4, 5). Oral administration of N-acetylcysteine has also an analgesic effect on nociceptive behavior in mice and laser-evoked pain in humans, since it can enhance endogenous activation of group II mGluRs (6, 7). Oral application of mGluR2/3 agonist prodrug results in a broad spectrum of analgesic actions in neuropathic, inflammatory, and visceral pain models (8). Morphological studies have shown that group II mGluRs are widely expressed in primary sensory neurons (9, 10, 11, 12). In periphery, group II mGluRs have also analgesic effects. Intraplantar injection of group II mGluR agonists reduces capsaicin- or prostaglandin E2–induced hyperalgesia in rodents (4, 5). Peripheral mGluR agonists can block hyperalgesia induced by inflammation and chemotherapy (13, 14). Peripheral injection of group II agonists inhibits peripheral nociceptor activity (15). Overexpression of mGluR2 in the dorsal root ganglia (DRG) has shown to be effective in inflammatory and neuropathic pain (16, 17, 18). In peripheral terminals of sensory neurons, group II mGluRs regulate transient receptor potential vanilloid type 1 (TRPV1) channels and tetrodotoxin-resistant sodium channels (5, 19, 20, 21). Peripheral group II mGluRs are therefore a promising therapeutic target for pain, although its mechanisms are not yet fully understood.

Acid-sensing ion channels (ASICs) are voltage-insensitive cation channels activated by extracellular pH drops (22, 23). There are currently at least six identified ASIC subunits encoded by four genes (24). Three subunits assemble into functional channels that can detect changes in extracellular pH. Of all ASIC subunit configurations, those containing ASIC3 are the most sensitive to protons (25). All ASICs except ASIC4 and ASIC5 are found in both DRG cell bodies and sensory terminals (26, 27). Among them, ASIC3 is the most prevalent ASIC subunit in DRG neurons, where it forms homotrimeric and heterotrimeric complexes and has emerged as a critical pH sensor (28, 29). Expression of ASIC3 gene is increased in DRG neurons after inflammation and nerve injury (29). Knockout or knockdown of ASIC3 reduces hyperalgesia in chronic neuropathic pain and inflammatory pain (29, 30). Tissue acidification occurs under various painful conditions such as inflammation, tissue injury, and ischemic stroke (24). ASICs, rather than TRPV1, play a major role in pain associated with tissue acidification, since the acid (up to pH 6.0)-induced pain is significantly alleviated by the nonselective ASIC inhibitor amiloride (29, 31). ASICs, especially peripheral ASIC3, are also a therapeutic target for pain (24, 28).

Although both group II mGluRs and ASICs are localized in DRG neurons and participate in various pain processes, it is still unclear whether there is a link between them. Herein, we report that the group II mGluR agonist LY354740 inhibited the electrophysiological activity of ASICs in rat DRG neurons through a mechanism that depended on G_i/o_ proteins and intracellular cAMP/protein kinase A (PKA) signaling pathway. LY354740 also relieved acid-induced nociceptive behaviors in rats by activating peripheral group Ⅱ mGluRs.

## Results

### LY354740 inhibits ASIC currents in rat DRG neurons

**Figure 1:**
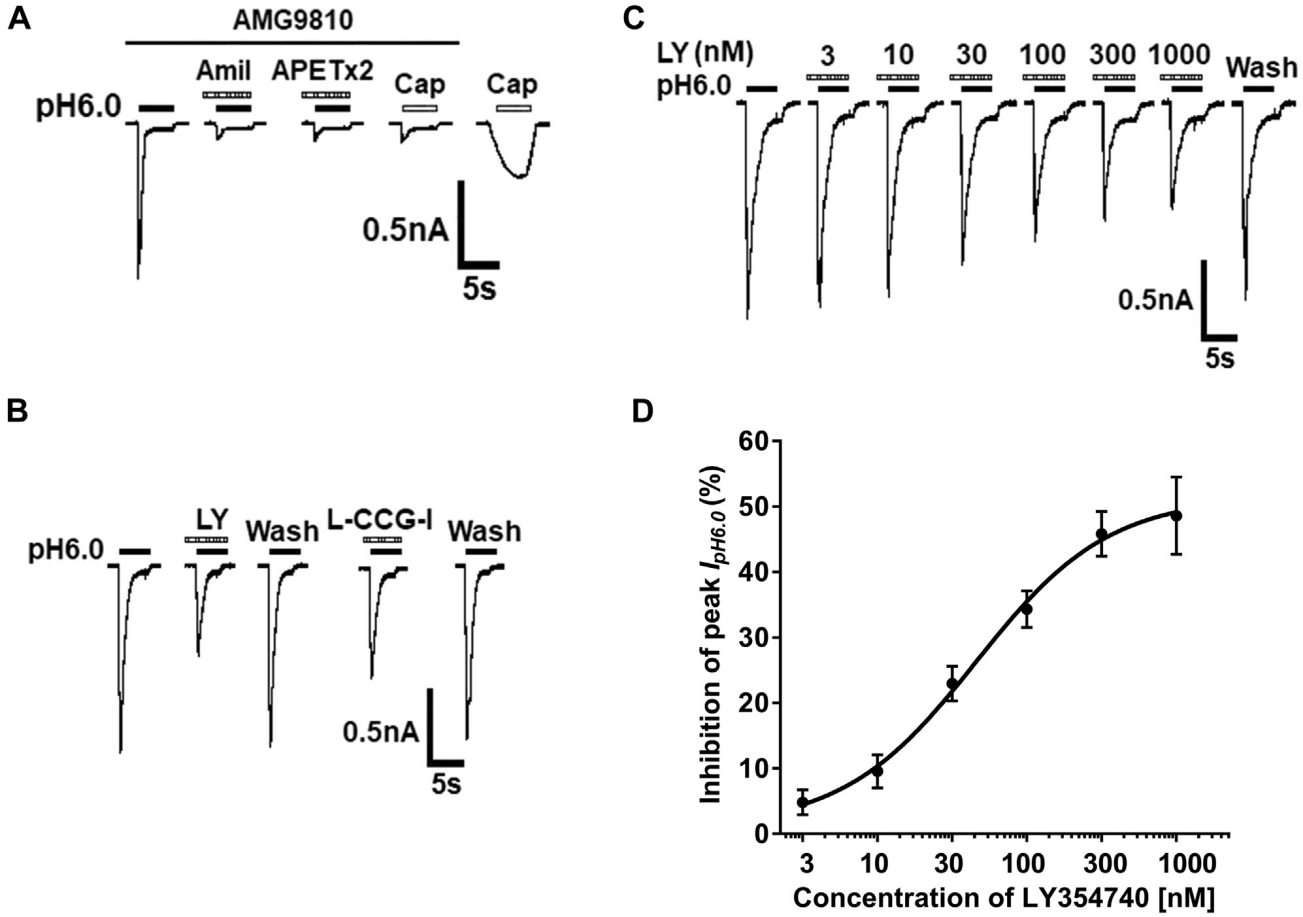
**LY354740 inhibits acid-sensing ion channel currents in rat dorsal root ganglion (DRG) neurons.***A*, under the conditions of external solution containing 5 μM AMG9810, an application of pH 6.0 acidic solution for 5 s caused a rapid inward current (I_pH6.0_) in a DRG neuron. The I_pH6.0_ was completely blocked by amiloride (Amil, 100 μM) and APETx2 (2 μM). Capsaicin (Cap, 100 nM) failed to induce any membrane currents in the presence of AMG9810, but it produced a slow desensitized inward current after washout of AMG9810 in the same cell. *B*, representative currents show that I_pH6.0_ was inhibited by either LY354740 (LY, 300 nM) or L-CCG-I (10 μM) and recovered after washout of them in the same DRG neuron. *C*, the sequential current traces illustrate the inhibition of I_pH6.0_ by different concentrations of LY354740 (LY, 3 nM - 1000 nM). Representative currents were recorded for more than 60 min in a representative DRG cell with membrane potential clamped at −60 mV. LY354740 was preapplied to recorded DRG cells for 3 min. *D*, the graph shows the concentration–effect curve of LY354740 on inhibition of I_pH6.0_ with an IC_50_ value of 37.67 ± 0.58 nM. Each point represents the mean ± SEM of 7 to 8 cells from 3 to 5 rats.

A 5-s exposure of pH 6.0 acidic solution can evoke a rapid inward current (I_pH6.0_) in the majority of DRG neurons (84.6%, 11/13 cells from 4 rats), even though acid-induced TRPV1 activation was blocked by addition of AMG9810 (5 μM) in external solution (Fig. 1*A*). In all DRG neurons response to pH 6.0 acid stimuli, most (72.7%, 8/11) of I_pH6.0_ were characterized by a fast inactivated inward current, followed by a smaller and nondesensitizing sustained current. The I_pH6.0_ with the characteristic was blocked by the broad-spectrum ASIC channel blocker amiloride (10 μM), also by ASIC3 blocker APETx2 (2 μM). Capsaicin (100 nM), a TRPV1 agonist, did not produce any membrane currents in all tested eight DRG neurons in the presence of AMG9810, while it evoked a slow desensitized inward current in the majority of DRG cells (71.4%, 5/8) after washout of AMG9810. Therefore, these acid-induced currents were identified as ASIC currents or ASIC3-like currents. In the present study, only ASIC3-like currents with a transient and sustained current component were used for the analysis. In addition, when pH 6.0 acidic solution was applied regularly for durations of 5 s with 5-min intervals, the I_pH6.0_ was stable for more than 60 min, and the change in amplitude was within 6.0%. Thus, we used this pattern of low pH applications in the following experiments.

In some neurons that recorded ASIC3-like currents, we observed that the amplitude of peak I_pH6.0_ was inhibited when the group Ⅱ mGluR agonist LY354740 (300 nM) or (2S,1′S,2′S)-2-(carboxycyclopropyl)glycine (L-CCG-I, 10 μM) was pretreated to DRG cells for 3 min prior to the next recording (Fig. 1*B*). The concentrations of 300 nM LY354740 and 10 μM L-CCG-I were higher than their half-maximal response (EC_50_) values of activated group Ⅱ mGluRs, which are less than 0.1 μM and 1 μM, respectively (32). The inhibitory effect of LY354740 and L-CCG-I on I_pH6.0_ disappeared after a 5-min washout and was reproducible in the same DRG neuron. In 7 of 12 DRG cells showing the ASIC3-like currents, preapplication of LY354740 (300 nM) and L-CCG-I (10 μM) decreased the amplitude of peak I_pH6.0_ by 44.14 ± 3.84% and 32.63 ± 3.27%, respectively. However, the other five cells showing the ASIC3-like currents were nonresponsive to LY354740 and L-CCG-I. In the present study, we established a cutoff value for the effects of LY354740 and L-CCG- I, which was a change in the amplitude of I_pH6.0_ exceeding 10%. Figure 1, *C* and *D* show the inhibition of I_pH6.0_ was dependent on the concentration of LY354740. The amplitude of peak I_pH6.0_ gradually decreased as concentration of LY354740 increased from 3 nM to 1000 nM in a recorded DRG neuron (Fig. 1*C*). Figure 1*D* shows the concentration–effect curve of LY354740 on I_pH6.0_ with an IC_50_ (half-maximal effective concentration) value of 37.67 ± 0.58 nM. The results suggested that LY354740 concentration-dependently inhibited ASIC currents.

**Figure 2:**
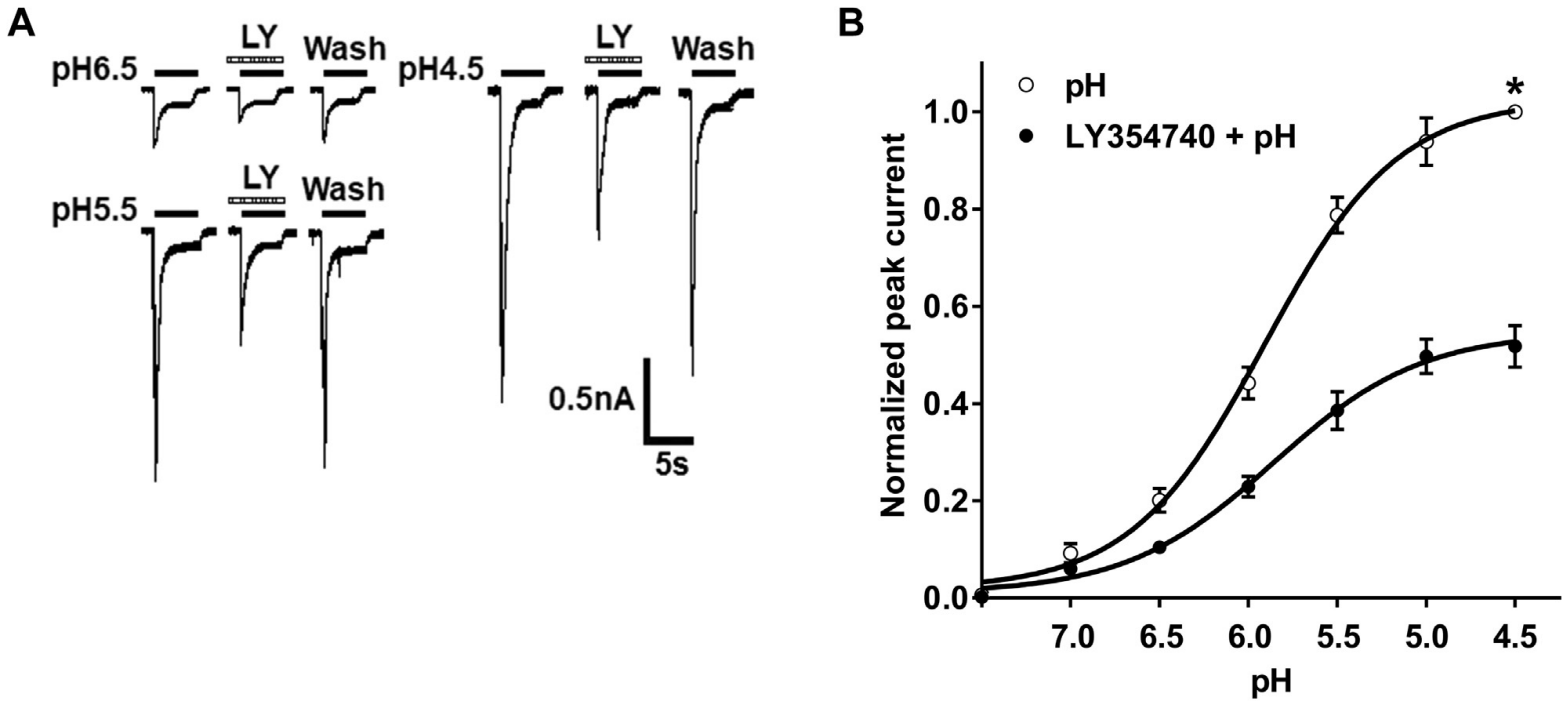
**LY354740 downwardly shifts the concentration–response curve for protons.***A*, representative current traces show preapplication of LY354740 (LY, 300 nM for 3 min) inhibited three acid-sensing ion channel currents evoked by acidic solution of pH 6.5, pH 5.5, and pH 4.5, respectively. *B*, the graph shows the concentration–response curve for protons was downwardly shifted by 300 nM LY354740 pretreatment, showing a decrease in the maximum response to protons. The curves were drawn according to the logistic equation I = I _max_/[1 + (10∧pH_0.5_/10∧pH) ^n^], where I is the normalized current response value, pH_0.5_ is pH for half-maximal activation, and n is the Hill coefficient. All current values from the same cell were normalized to the current response, which was induced by pH 4.5 applied alone without LY354740 pretreatment (marked with *asterisk*). Each point represents the mean ± SEM of 7 to 8 cells from 3 to 5 rats.

We then investigated the effects of LY354740 on ASIC currents evoked by different acidic pH values. Figure 2*A* shows that preapplication of LY354740 (LY, 300 nM for 3 min) decreased the amplitudes of three ASIC currents, which were evoked by acidic solution of pH 6.5, pH 5.5, and pH 4.5, respectively. Figure 2*B* shows that LY354740 downwardly shifted the concentration–response to protons, which wase plotted through a series of different acidic pH solutions. Preapplication of LY354740 (300 nM for 3 min) to recorded DRG cells resulted in a decrease of 48.21 ± 4.27% in the maximal current response (I_pH4.5_) of curve, which was induced by acidic solution of pH 4.5 in the absence of LY354740. However, LY354740 did not change pH_0.5_ (pH for half-maximal activation) values, showing no significant difference in the pH_0.5_ values between the two curves (pH: pH_0.5_ = 5.9 ± 0.1; LY354740 + pH: pH_0.5_ = 5.8 ± 0.2; *p* > 0.1, post hoc Bonferroni test). In addition, there was no significant difference in the slope or Hill coefficient between the two curves (pH: n  =  1.2 ± 0.2; LY354740 + pH: n  =  1.1 ± 0.3; *p* > 0.1, post hoc Bonferroni test). These results indicated that LY354740 regulated the maximum response of ASICs to protons but not their acid sensitivity.

### Group Ⅱ mGluRs and downstream intracellular signaling underlie the inhibition of ASIC currents by LY354740

**Figure 3:**
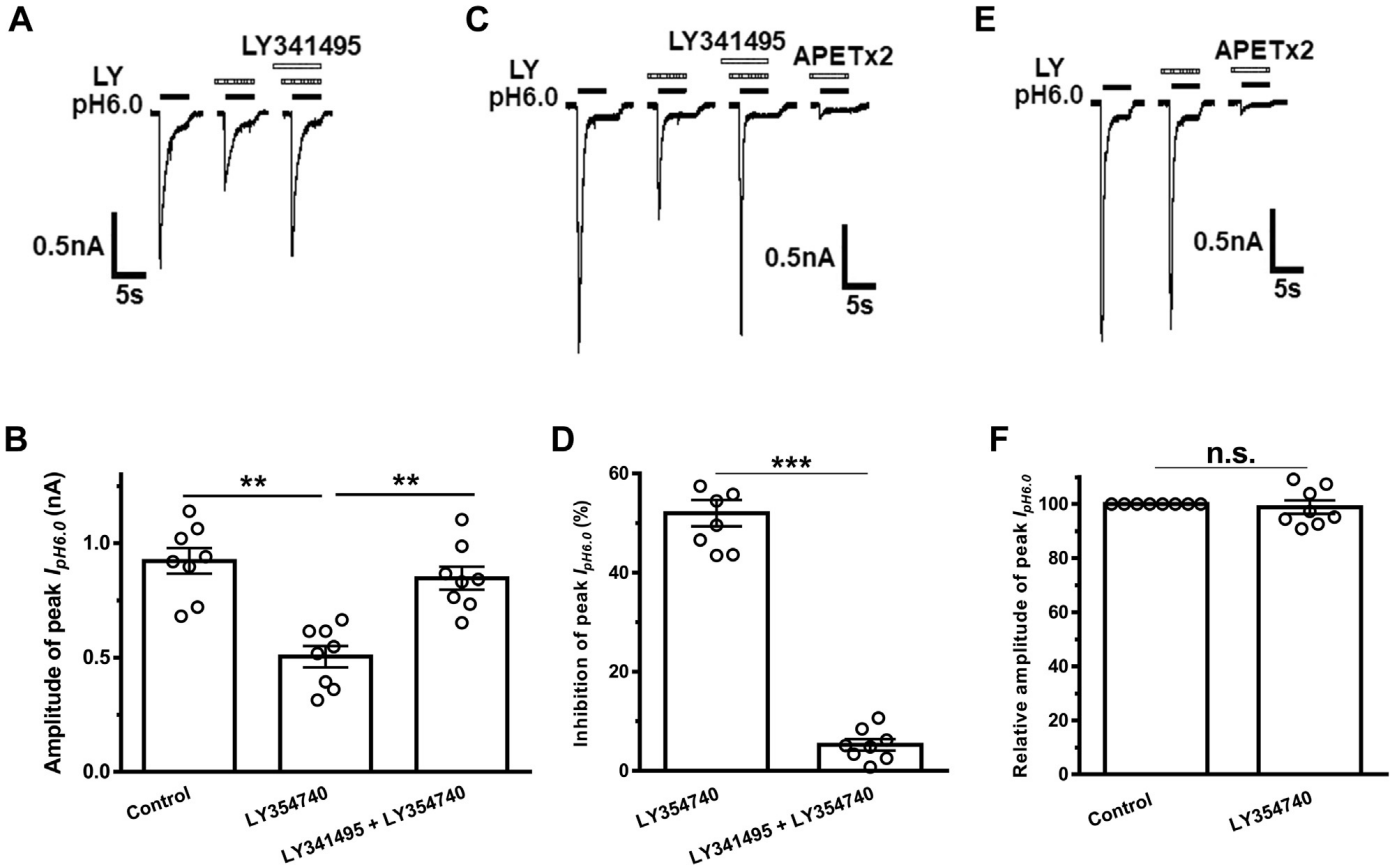
**Group Ⅱ mGluRs participate in the inhibition of acid-sensing ion channel (ASIC) currents by LY354740.** Representative current traces in (*A*) show I_pH6.0_ was recorded in a dorsal root ganglion neuron under conditions of control, treatment with LY354740 (300 nM for 3 min), and cotreatment with LY354740 treatment (300 nM for 3 min) and the group Ⅱ mGluR antagonist LY341495 (500 nM for 4 min), respectively. The bar graph in (*B*) shows that amplitude of peak I_pH6.0_ was inhibited by 300 nM LY354740 preapplied alone for 3 min, and the inhibitory effect of LY354740 was blocked by 500 nM LY341495 in dorsal root ganglion neurons. ∗∗*p* < 0.01; Bonferroni post hoc test, n = 8 cells from 5 rats in each column. Current traces in (*C*) and the bar graph in (*D*) show I_pH6.0_ was inhibited by preapplication of LY354740 (300 nM for 3 min) but not by cotreatment with LY354740 (300 nM for 3 min) and LY341495 (500 nM for 4 min), in CHO cells cotransfected with mGluR2 and ASIC3. I_pH6.0_ was blocked by APETx2 (2 μM). ∗∗∗*p* < 0.001, unpaired *t* test. n = 8 cells in each column. *E* and *F* LY354740 treatment (300 nM for 3 min) had no effect on the I_pH6.0_ in ASIC3-transfected CHO cells without expression of mGluR2. I_pH6.0_ was blocked by APETx2 (2 μM). Currents were normalized to the control (100%). ns, not significant, unpaired *t* test. n = 8 cells in each column.

We studied whether group Ⅱ mGluRs mediated the inhibition of ASIC currents by LY354740. LY341495, a group Ⅱ mGluR antagonist, was also coapplied with LY354740 to recorded DRG cells. As shown in Figure 3, *A* and *B*, LY354740 (LY, 300 nM for 3 min) pretreatment alone to recorded DRG cells resulted in a significant decrease in the amplitude of peak I_pH6.0_ (from 0.96 ± 0.06 nA to 0.50 ± 0.05 nA; *p* < 0.01, one-way ANOVA followed by post hoc Bonferroni test, n = 8 cells from 4 rats). In contrast, the amplitude of peak I_pH6.0_ was 0.85 ± 0.05 nA in eight DRG cells pretreated with both LY341495 (500 nM for 4 min) and LY354740 (LY, 300 nM for 3 min), which was no different from 0.96 ± 0.06 nA in control conditions. The results indicated that LY354740 inhibited ASIC currents by activating group Ⅱ mGluRs in DRG neurons.

To further verify involvement of group Ⅱ mGluRs in LY354740-induced inhibition of acid-evoked currents, ASIC3 and mGluR2 were coexpressed in Chinese hamster ovary (CHO) cells. We recorded I_pH6.0_ in these cotransfected cells, which was completely blocked by APETx2 (2 μM), suggesting ASIC3 channel activation. Similar to that observed in DRG neurons, LY354740 pretreatment (LY, 300 nM for 3 min) also significantly inhibited the ASIC3 currents in CHO cells coexpressing ASIC3 and mGluR2 (Fig. 3, *C* and *D*). The inhibitory effect of LY354740 on ASIC3 currents was also blocked by preincubation of LY341495 (500 nM for 4 min; *p* < 0.01, unpaired *t* test, n = 8; Fig. 3, *C* and *D*). In contrast, LY354740 pretreatment (LY, 300 nM for 3 min) failed to change ASIC3 currents in CHO cells expressing ASIC3 alone, but not expressing mGluR2 (*p* ＞ 0.05, unpaired *t* test, n = 8; Fig. 3, *E* and *F*). These results indicate that mGluR2 mediated LY354740-induced inhibition of ASIC3 currents.

**Figure 4:**
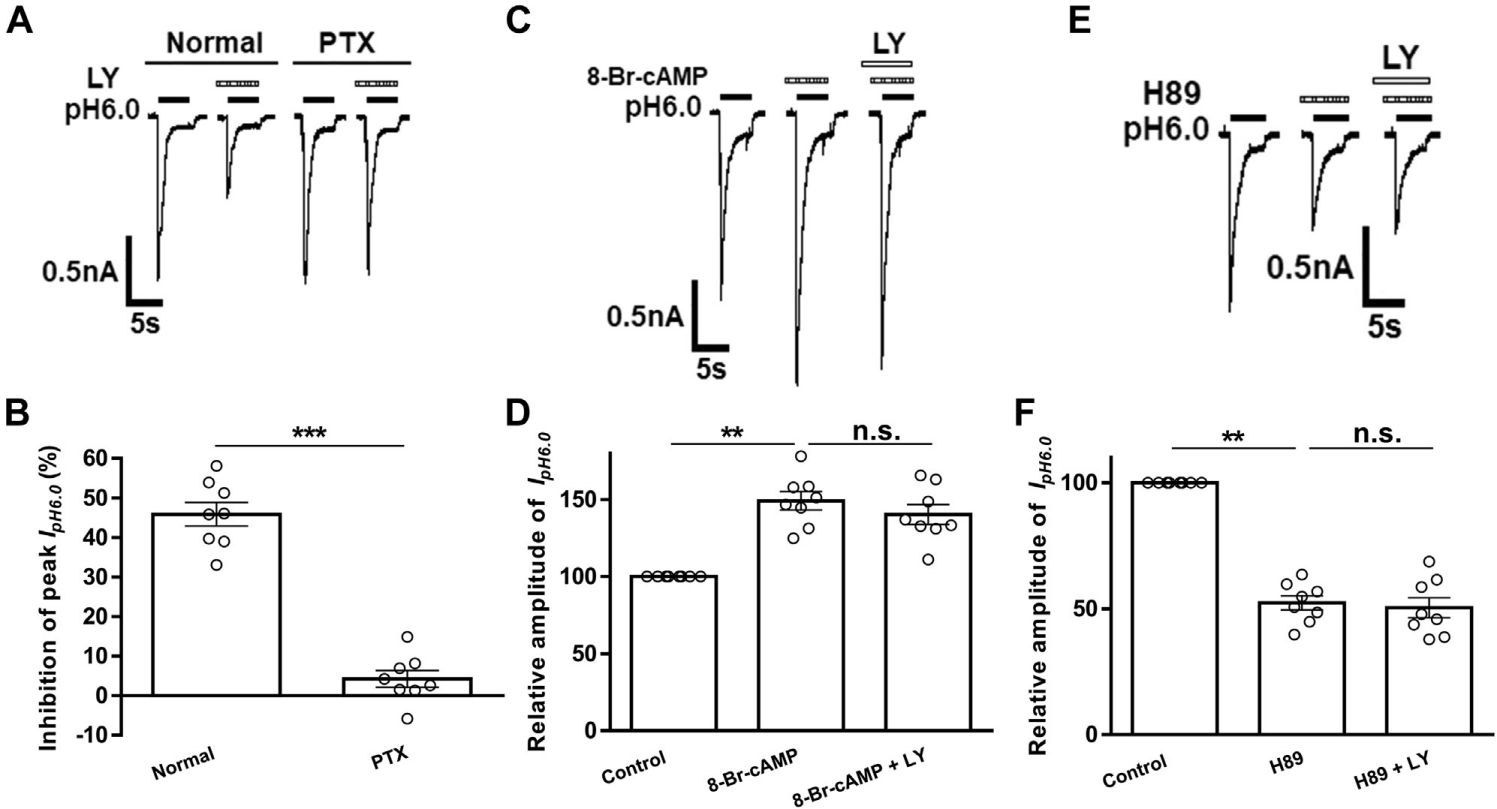
**G**_**i/o**_**proteins and protein kinase A signaling are involved in group Ⅱ mGluR-induced inhibition of acid-sensing ion channel currents.** The current traces in (*A*) and the bar graph in (*B*) show LY354740 (LY, 300 nM for 3 min) significantly inhibited I_pH6.0_ in dorsal root ganglion neurons under normal internal solution conditions but not under conditions of recording pipettes filled with pertussis toxin (PTX) (1 μg/ml)-containing internal solution. The current traces in (*C*) and the bar graph in (*D*) show that I_pH6.0_ was enhanced by preincubation with 8-Br-cAMP (1 mM), a membrane permeable cAMP analogue, for 5 min. LY354740 (LY, 300 nM for 3 min) failed to inhibit I_pH6.0_ in DRG cells treated with 8-Br-cAMP. The current traces in (*E*) and the bar graph in (*F*) show preincubation of 0.3 μM H89, a protein kinase A inhibitor, for 5 min mimicked the inhibitory effect of LY354740 on I_pH6.0_. But LY354740 (LY, 300 nM for 3 min) did not cause a further inhibition of I_pH6.0_ in H89-treated dorsal root ganglion cells. ∗∗*p* < 0.01, ∗∗∗*p* < 0.001, n.s., not significant. Bonferroni post hoc test. n = 8 cells from 4 or 5 rats in each column.

We further investigated the downstream intracellular signaling mechanisms underlying the inhibition of ASIC currents by group Ⅱ mGluR activation. Group Ⅱ mGluRs couple to G_i/o_ proteins, and therefore their activation promotes the inhibition of adenylate cyclase activity and the cAMP/PKA pathway (33, 34). First, unlike an inhibition of 45.82 ± 3.42% in normal internal solution conditions, the group Ⅱ mGluR agonist LY354740 (LY, 300 nM for 3 min) failed to decrease the amplitude of peak I_pH6.0_ after pertussis toxin (PTX, 1 μg/ml), an inhibitor of G_i/o_ proteins, was applied internally to recorded DRG neurons (*p* < 0.001, unpaired *t* test, n = 8 cells from 5 rats), suggesting that PTX prevented the decrease of I_pH6.0_ amplitude induced by LY354740 (Fig. 4, *A* and *B*). Second, 8-Br-cAMP (1 mM), a membrane-permeable cAMP analogue, was preapplied to recorded DRG cells, resulting in an increase of 149.17 ± 5.89% in relative amplitudes of I_pH6.0_ (*p* < 0.01, one-way ANOVA followed by post hoc Bonferroni test, n = 8 cells from 4 rats; Fig. 4, *C* and *D*). In the presence of 8-Br-cAMP (1 mM), preapplication of LY354740 (LY, 300 nM for 3 min) failed to decrease the amplitude of peak I_pH6.0_ (*p* > 0.1, compared with 8-Br-cAMP treatment alone, one-way ANOVA followed by post hoc Bonferroni test, n = 8 cells from 4 rats; Fig. 4, *C* and *D*). Third, preapplication of the membrane-permeable PKA inhibitor H89 (0.3 μM) to recorded DRG cells mimicked the effect of LY354740, producing an inhibition of 47.69 ± 2.80% in the amplitude of peak I_pH6.0_ (*p* < 0.01, one-way ANOVA followed by post hoc Bonferroni test, n = 8 cells from 4 rats; Fig. 4, *E* and *F*). But LY354740 (LY, 300 nM for 3 min) did not further inhibit I_pH6.0_ in H89-treated DRG cells (*p* > 0.1, compared with H89 treatment alone, one-way ANOVA followed by post hoc Bonferroni test, n = 8 cells from 4 rats; Fig. 4, *E* and *F*). These results suggested that group Ⅱ mGluR activation inhibited ASIC currents via a G_i/o_ protein and intracellular cAMP/PKA signaling pathway.

### LY354740 decreases acid-triggered membrane excitability in rat DRG neurons

**Figure 5:**
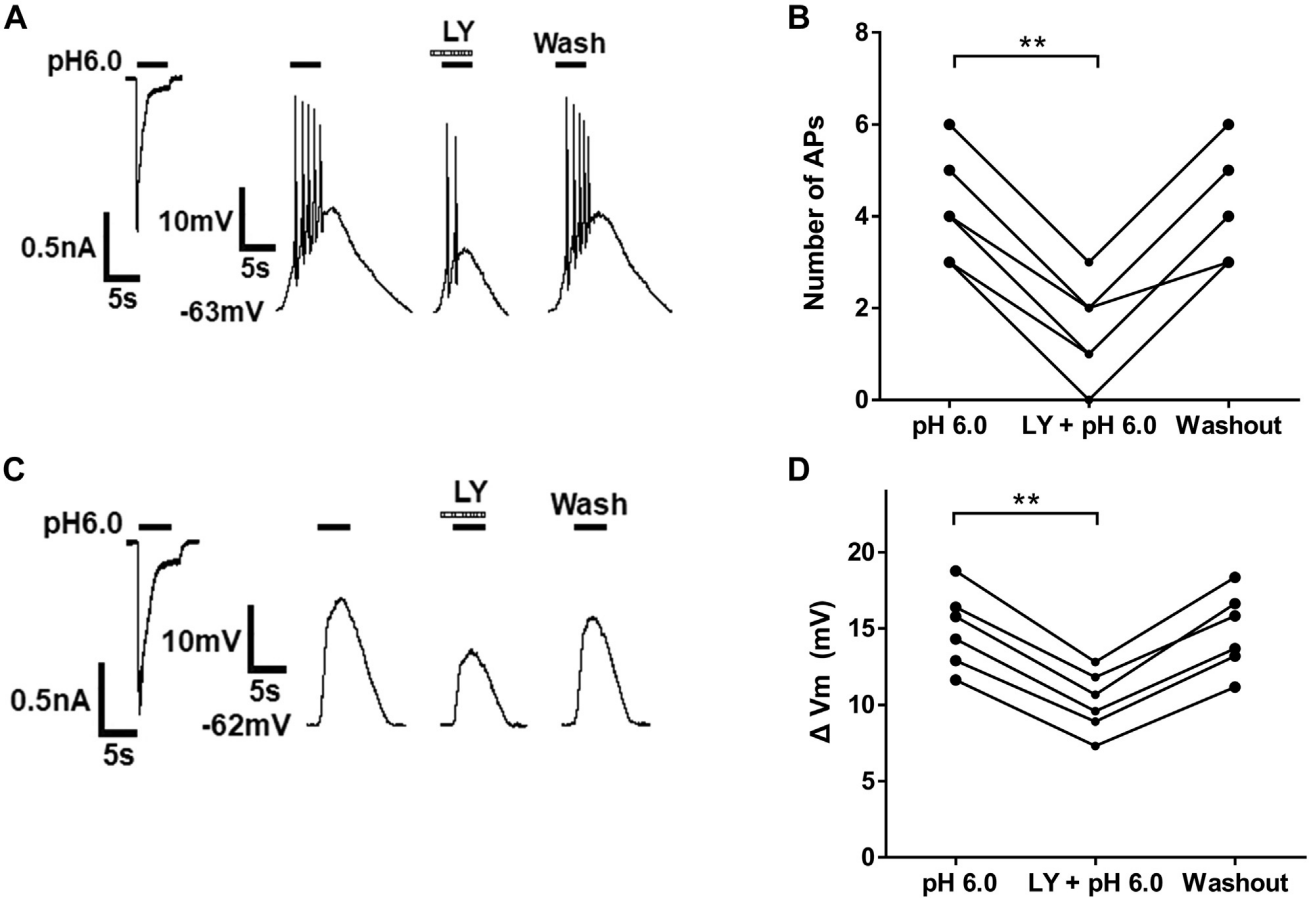
**LY354740 decreases proton-evoked membrane excitability in rat dorsal root ganglion (DRG) neurons.** Original traces in (*A*) show an acid stimulus of pH 6.0 caused an inward current and action potentials (APs) in the same DRG cell under voltage-clamp and current-clamp conditions, respectively. The external solution contained 5 μM AMG9810 to block TRPV1 activation. Original APs in (*A*) were recorded before and during application of LY354740 (LY, 300 nM for 3 min) and after washout of LY354740 in a DRG cell. Data in (*B*) show preapplication of LY354740 (LY, 300 nM for 3 min) significantly decreased the number of APs evoked by pH 6.0 in six DRG cells from three rats. The number of APs recovered after washout of LY354740. ∗∗*p* < 0.01, paired *t* test. Original traces in (*C*) show an acid stimulus of pH 6.0 caused an inward current under voltage-clamp mode and depolarization of membrane potentials under current-clamp mode in the same DRG cell. The external solution contained AMG9810 (5 μM) and tetrodotoxin (1 μM) to block TRPV1 activation and Na^+^ channel–mediated APs, respectively. Original traces in (*C*) and data in (*D*) show preapplication of LY354740 (LY, 300 nM for 3 min) significantly decreased membrane depolarization (ΔVm) evoked by pH 6.0 in six DRG cells from three rats. The ΔVm recovered after washout of LY354740. ∗∗*p* < 0.01, paired *t* test.

To investigate the effect of LY354740 on membrane excitability triggered by acidic stimuli, we recorded I_pH6.0_ and action potentials (APs) under voltage-clamp and current-clamp conditions, respectively, which were triggered by the acidic stimuli of pH 6.0 in the presence of 5 μM AMG9810 in the same DRG cells (Fig. 5*A*). Consistent with the inhibitory effect of LY354740 on ASIC currents under voltage-clamp conditions, LY354740 also decreased the bursts of acid-evoked APs. Figure 5*B* shows that preapplication of LY354740 (LY, 300 nM for 3 min) decreased the number of APs evoked by acidic stimuli of pH 6.0 from 4.17 ± 0.48 to 1.50 ± 0.43 in six DRG cells (*p* < 0.01, paired *t* test, n = 6 cells from 3 rats). The number of APs recovered to the level before LY354740 treatment after the 5-min washout of LY354740. To further record acid-evoked membrane potential depolarization (ΔVm) in DRG cells, AMG9810 (5 μM) and tetrodotoxin (1 μM) were contained in the external solution to block acid-induced TRPV1 activation and Na^+^ channel–mediated APs, respectively. Figure 5, *C* and *D* show that preapplication of LY354740 (LY, 300 nM for 3 min) decreased the magnitude of ΔVm triggered by acidic stimuli of pH 6.0 from 14.97 ± 1.05 mV to 10.16 ± 0.82 mV (paired *t* test, *p* < 0.01, n = 6 cells from 3 rats). These results indicate that LY354740 decreased acid-triggered membrane excitability in rat DRG neurons.

### Peripheral group Ⅱ mGluR activation relieves acid-induced nociceptive behaviors in rats

**Figure 6:**
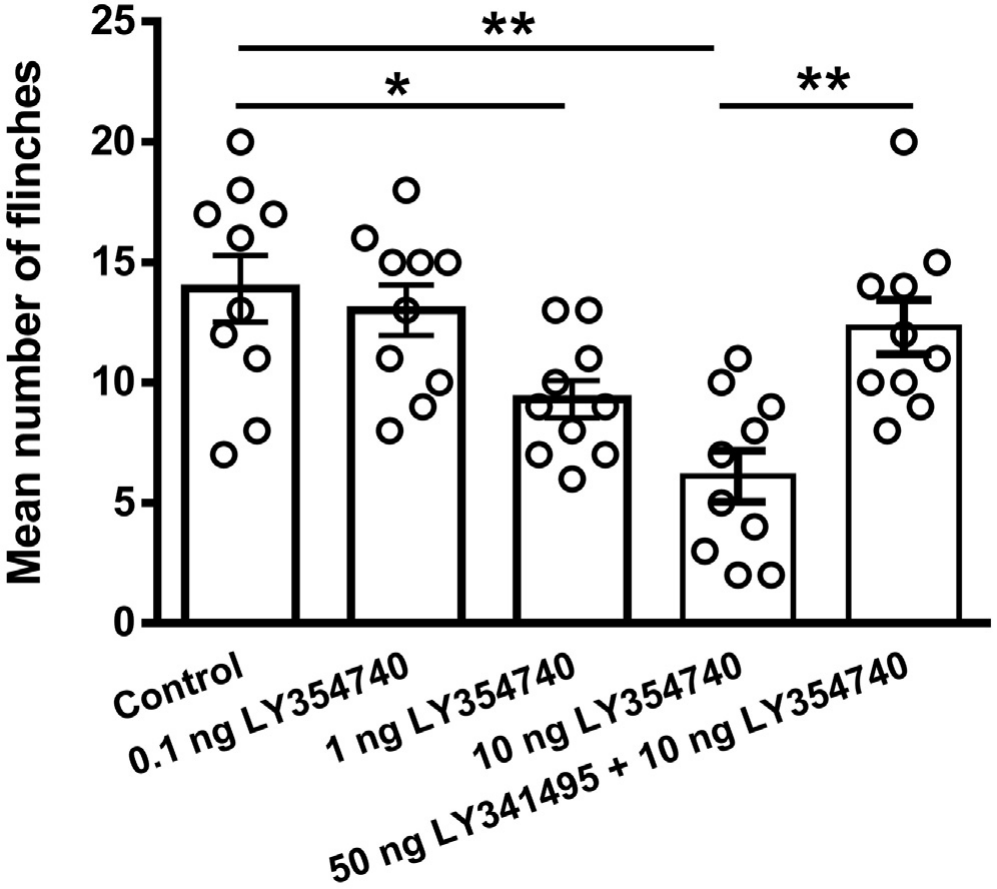
**LY354740 relieves acid-induced nociceptive behaviors in rats.** Intraplantar injection of acetic acid (1%, 50 μl) resulted in significant flinching behaviors even in the presence of the TRPV1 inhibitor AMG9810 (10 μM). Intraplantar pretreatment of LY354740 (0.1, 1, and 10 ng) dose-dependently reduced the number of acid-induced flinching. Analgesia of LY354740 (10 ng) on the flinching behaviors was prevented by cotreatment of the group Ⅱ mGluR antagonist LY341495 (50 ng). ∗*p* < 0.05, ∗∗*p* < 0.01; Bonferroni post hoc test. Each column represents the mean ± SEM of 10 rats.

Intraplantar injection of low-pH solution produces intense flinch/shaking responses in rats by activating ASICs (29, 35). Finally, we observed whether the group Ⅱ mGluR agonist LY354740 affected acid-induced nociceptive behaviors *in vivo*. Rats displayed spontaneous flinching/shaking responses when acetic acid (1% v/v, 50 μl) was injected into the hind paws, even with blockade of TRPV1 by AMG 9810 (10 μM). Pretreatment with LY354740 (0.1, 1, and 10 ng in 50 μl) in ipsilateral hind paws dose-dependently relieved the acid-induced nociceptive behaviors (*p* < 0.05 and 0.01, one-way ANOVA followed by post hoc Bonferroni test, n = 10 rats; Fig. 6). In contrast, after receiving injection of the group Ⅱ mGluR antagonist LY341495 (50 ng) + LY354740 (10 ng), these rats displayed nociceptive behaviors that were no different from those seen in rats injected with acetic acid alone, suggesting LY341495 blocked the relieving effect of LY354740 on acid-induced nociceptive behaviors (*p* < 0.01, one-way ANOVA followed by post hoc Bonferroni test, n = 10 rats; Fig. 6). In addition, injection of LY354740 (10 ng in 50 μl) in one hind paw did not change nociceptive behaviors induced by acetic acid injected in the other hind paw (data not shown). Intraplantar injection of LY354740 (10 ng in 50 μl) alone or LY341495 (50 ng in 50 μl) alone caused little or no behavioral response (mean number of flinches: 1.80 ± 0.42 and 1.90 ± 0.38, respectively; n = 10) that was not significantly different from vehicle injection (mean number of flinches:1.50 ± 0.43; n = 10). The results suggested that activation of peripheral group Ⅱ mGluRs by LY354740 relieved acid-induced nociceptive behaviors in rats.

## Discussion

The present data demonstrated that activation of group Ⅱ mGluRs by LY354740 reduced acid-evoked ASIC currents and APs in rat DRG neurons. LY354740 indirectly inhibited the electrophysiological activity of ASICs through PTX-sensitive G_i/o_ proteins and cAMP/PKA signaling cascade. LY354740 also inhibited ASIC3 currents in CHO cells coexpressing mGluR2 and ASIC3 but not in cells expressing ASIC3 alone. LY354740 relieved acid-induced nociceptive behaviors in rats by activating peripheral group Ⅱ mGluRs.

In the present study, the acid-induced currents were ASIC currents rather than TRPV1 currents, since they were completely blocked by the ASIC channel blocker amiloride. Furthermore, most of these ASIC currents were ASIC3-like currents, which were sensitive to the ASIC3 blocker APETx2 and characterized by a fast inactivated inward current, followed by a smaller and nondesensitizing sustained current (29). We observed that the Hill coefficient for the ASIC3-like current activation in DRG neurons was lower than the slope or Hill coefficient of 2.36 in homomeric ASIC3-expressing CHO cells (36). One explanation may be that the present ASIC3-like currents were due to activation of ASIC3-containing heteromeric channels rather than ASIC3 homotrimers. This was consistent with the morphological evidence that ASICs are expressed in DRG neurons, with ASIC3 being the most prevalent ASIC subunit (26, 27, 29). The present study showed that two group Ⅱ mGluR agonists, LY354740 and L-CCG-I, had inhibitory effects on ASIC currents in rat DRG neurons. LY354740 decreased the maximum response of ASICs to proton, but it did not change the acid sensitivity of ASICs. Consistent with results in the voltage-clamp experiments, LY354740 also decreased the number of APs and the magnitude of membrane potential depolarization evoked by acidic pH solution under the current-clamp conditions, suggesting a decreased membrane excitability of rat DRG neurons.

LY354740 inhibited ASICs through group Ⅱ mGluRs, since the inhibitory effects of LY354740 on ASIC currents and acid-induced nociceptive behaviors were completely blocked by LY341495, a group Ⅱ mGluR antagonist. Immunoreactivity of group II mGluRs has been observed in rat DRG neurons (9, 12). The present experiments in DRG neurons cannot distinguish between mGluR2 and mGluR3 subtypes mediating the effects of LY354740. Since mRNA for the mGluR3 subtype was not found in DRG (37), it is highly likely that the mGluR2 subtype was responsible for the effects of LY354740 on ASIC currents. Furthermore, we observed that LY354740 decreased ASIC3 currents in CHO cells that coexpressed ASIC3 and mGluR2 but not in ASIC3-transfected CHO cells without expression of mGluR2, suggesting that the mGluR2 subtype mediated the inhibition of ASIC3 current by LY354740. We believed that the inhibitory effect of LY354740 on ASIC currents was indirect, which only occurred in these DRG neurons coexpressing group II mGluRs and ASICs, although further morphological evidence was needed.

Group II mGluRs belong to the G_i/o_ members of the G protein–coupled family; their activation inhibits CA and the cAMP/PKA pathway and lowers the levels of cAMP (33, 34). The present results demonstrated that G_i/o_ proteins and intracellular cAMP/PKA signaling were involved in the link between group II mGluRs and ASICs in rat DRG neurons. First, inhibitory effect of LY354740 on ASIC currents disappeared after G_i/o_ proteins were blocked by PTX. Second, LY354740-induced inhibition of ASIC currents was lack in DRG neurons pretreated with the cAMP analogue 8Br-cAMP. Finally, LY354740 failed to further reduce ASIC currents after intracellular PKA inhibition; however, the inhibitory effect of LY354740 on ASIC currents was mimicked by the PKA inhibitor H-89. These results were consistent with findings that ASICs are the downstream targets of intracellular cAMP/PKA signaling. G_i/o_ protein–coupled 5-HT_1D_ receptor activation inhibits ASIC currents in rat trigeminal ganglion neurons through a cAMP-dependent signaling pathway (38). Our previous studies indicate that cAMP/PKA signaling is involved in negative regulation of ASIC activity by other G_i/o_ protein–coupled receptors, such as μ-opioid receptors and CB1 cannabinoid receptors, in rat DRG neurons (39, 40). These studies provide further support that LY354740 suppressed ASIC currents in an intracellular cAMP/PKA signaling-dependent manner. ASIC1 isoforms are also expressed in rodent sensory neurons and appear to be more highly expressed than ASIC3 in human DRG neurons (26, 41). PKA has also been shown to phosphorylate the C terminus of ASIC1 (42). Suppression of PKA phosphorylation of ASIC1 has been found to decrease the amplitude of ASIC1 currents in cortical neurons (43). Speculatively, group II mGluR activation may also have an inhibitory effect on ASIC1 currents, including in human DRG neurons, through G_i/o_ proteins and cAMP/PKA signaling cascade.

It has been reported that activation of peripheral group II mGluRs reduces nociceptive behaviors in inflammatory states but not in nonsensitized states (44). The group II mGluR agonist also attenuates capsaicin-induced nociceptive behaviors in rats (20). We extend these findings, demonstrating that peripheral pretreatment of the group II mGluR agonist LY354740 dose-dependently relieved the acid-evoked nociceptive behaviors in rats. Immunoreactivity for the mGluR2 subtype has been identified in terminal fibers within the footpad epidermis (15). The present study showed that LY354740 exerted its antihyperalgesic effect by acting directly on local group II mGluRs localized on nociceptors, since its effect was significantly blocked by intraplantar pretreatment of LY341495, a group Ⅱ mGluR antagonist. Our results did not exclude that peripheral activation of group II mGluRs relieved acid-induced nociceptive behaviors through additional mechanisms, such as modulation of sodium channels and potassium channels. But we believed that inhibition of ASICs by LY354740 contributed to the relief of acid-evoked nociceptive behaviors, at least in part.

Tissue acidosis commonly develops in a variety of painful conditions, resulting in a local decrease in pH value. ASICs, especially ASIC3, localized on peripheral nociceptors can detect the pH change and respond to acidosis (45). Exposure of ASICs to an acidic pH causes cation influx, neuronal depolarization, and action potential firing in peripheral nociceptors, resulting in acid-induced nociceptive behaviors (46). The acid-evoked firing and nociception may be mediated by ASIC3 or ASIC3-containing channels, since they are blocked by pretreatment with APETx2 (36). However, we cannot exclude the involvement of sodium channels, since APEXx2 inhibits also voltage-gated sodium channels at similar concentrations (47, 48). When nociceptors are activated, peripheral glutamate is released (49, 50). Glutamate was recently shown to have a potentiating effect on ASIC1a (51). Our previous studies have shown that glutamate can activate peripheral group I mGluRs and then sensitizes ASICs (52). The present results indicated that the released glutamate inhibited the activity of ASICs through activation of peripheral group II mGluRs. These results indicated that glutamate can be directly linked to ASICs or indirectly linked to ASICs through group I and group II mGluRs, which may provide a homeostatic mechanism to prevent excessive acid signaling through ASICs. It has been reported that basal glutamate levels in hind-paw skin range from 1.2 to 1.9 μM and increase approximately 3-fold in formalin test (49, 50). Glutamate has higher potency at group II mGluRs (with the EC_50_ value as low as 0.04–0.3 μM) than at group I mGluRs (with the EC_50_ value of 1 μM) (32) whereas it takes more than 100 μM glutamate to directly enhance ASIC1a (51). Thus, group II mGluRs represent an appealing therapeutic target under pathological conditions involving ASICs. Clinically, side effects remain a challenge in group II mGluR agonist applications, since group II mGluRs are also widely expressed in the central nervous system. Limiting a group II mGluR drug to the periphery may reduce the side effects (53, 54). Our results indicated activating peripheral group II mGluRs by LY354740, even with local application, can also relieve acid-evoked pain by inhibiting ASICs in primary sensory neurons. Thus, peripheral administration of selective group II agonists may be potent therapeutic agents for treatment of pain, at least for pain associated with tissue acidification.

In summary, our results suggested peripheral group II mGluR activation attenuated ASIC-mediated electrophysiological activity and pain, revealing a novel peripheral mechanism for analgesics targeting peripheral group II mGluRs.

## Experimental procedures

### Preparation of DRG neurons

All experimental protocols were approved by the Animal Research Ethics Committee of Hubei University of Science and Technology (2016-03-005). A female Sprague-Dawley rat (5–6 weeks old) at a time was anesthetized and sacrificed. The DRGs of lumbar segments 4 to 6 were removed and chopped. The minced ganglia were transferred to a test tube containing Dulbecco's modified Eagle's medium and incubated with shaking for 25 to 30 min at 35 °C. The incubation solution contained 1.0 mg/ml of collagenase, 0.5 mg/ml of trypsin, and 0.1 mg/ml of IV DNase. Trypsin digestion was terminated by adding1.25 mg/ml of soybean trypsin inhibitor. The cells were cultured for 12 to 24 h at 37 °C in Dulbecco's modified Eagle's medium containing nerve growth factor (100 ng/ml) and fetal bovine serum (10%).

### Electrophysiological recordings

Electrophysiological experiments were performed as described (55). A MultiClamp-700B amplifier and Digidata-1440A A/D converter (Axon Instruments) were used for the whole-cell patch clamp recordings. The isolated DRG neurons were transferred to a 35-mm culture dish and kept in a normal external solution for at least 60 min before electrophysiological recordings. The external solution contained the following (in mM): 150 NaCl, 5 KCl, 2 MgCl_2_, 2.5 CaCl_2_, 10 Hepes, and 10 D-glucose. The pH and osmolarity were adjusted to 7.4 with NaOH and 330 mOsm/L with sucrose, respectively. The low pH value was prepared with HCl and external solution that contained 10 mM 4-morpholineethanesulfonic acid (MES) instead of 10 mM Hepes. The recording pipettes were pulled using a Sutter P-97 puller (Sutter Instruments), whose resistance was in the range of 3–6 MΩ. The micropipette solution contained the following (in mM): 140 KCl, 2 MgCl_2_, 11 EGTA, 10 Hepes, 4 ATP, and 0.3 Na_2_GTP. The pH and osmolarity were adjusted to 7.2 with KOH and 310 mOsm/L with sucrose, respectively. After gigaseal formation the pipette capacitance mediated current transients were compensated, and then the membrane beneath the pipette was ruptured by suction to form whole cell recording configuration. Then whole cell capacitance compensation was done, after which series resistance was compensated by 70 to 80%. The recorded currents were low-pass filtered at 2 kHz and sampled at 10 kHz. Only small and medium-sized nociceptive DRG cells (15–40 μm in diameter) were used for the electrophysiological recordings. The membrane potential of the recorded cells was clamped at −60 mV unless otherwise stated. Current-clamp recordings were carried out only in DRG cells whose resting membrane potentials were less than −50 mV.

### Cell transfection

Rat ASIC3 and mGluR2 cDNAs were used for heterologous expression in CHO cells as described (36). In brief, CHO cells were transiently transfected with HilyMax liposome transfection reagent. The cDNA ratio of ASIC3 and mGluR2 was 1:1, if they were cotransfected into the same CHO cell. To identify the positive transfected cells, the ASIC3 cDNA plasmid contained the coding sequence of green fluorescent protein. CHO cells were cultured in F-12 nutrient mixture containing gluta-MAXTM-1 (1%) and fetal bovine serum (10%). Electrophysiological recordings were carried out 24 to 48 h after transfection.

### Drug application

In the experiment, drugs included hydrochloric acid, LY354740, LY341495, L-CCG- I, 8-Br-cAMP, H-89, amiloride, APETx2, capsaicin, and AMG 9810. They were obtained from Sigma Chemical Co. The pH value of all working drugs was adjusted to 7.4 with NaOH and external solution containing 10 mM MES. Each working drug was stored in a series of independent reservoirs and applied by gravity. The distance was ∼30 μm between drug exit and recorded neurons. To block G-protein and intracellular signal, some antagonists or blockers were dissolved in the internal solution and applied for intracellular dialysis through recording patch pipettes as described (56). To ensure that dialysis drugs are infused into the cell interior, current recording was performed 30 min after cell membrane rupture. To purify ASIC activation, AMG9810 (5 μM) was added to the extracellular solution to block acid-induced TRPV1 activation.

### Nociceptive behavior induced by acetic acid in rats

Considering the gender differences in pain caused by acidosis in our previous reports, only female rats were used to study in the current experiment (57). Rats were first habituated for 30 min in a Plexiglas chamber during the nociceptive behavioral experiment. The rats received two intraplantar injections, each with a volume of 50 μl. For the first time, rats in five different groups (n = 10/group) injected with either 50 μl of 10 μM AMG 9810 + vehicle, a 50 μl of cocktail containing10 μM AMG 9810 + different doses (0.1, 1, and 10 ng) of LY354740, or 50 μl of cocktail containing AMG 9810 + 50 ng LY341495 + 10 ng LY354740. After 10 min, another experimenter injected 50 μl of acetic acid solution (1% v/v, pH value was adjusted to 6.0 with NaOH and external solution containing 10 mM MES) into the ipsilateral hind paws and tested the nociceptive behavior. The assessor of the behavioral measures was blinded to the prior treatment conditions. Nociceptive behavior (*i.e.*, number of flinching, shaking, and licking) was counted within 5 min after injection. In one group, acetic acid was injected into one hind paw and 10 ng LY354740 was injected into the contralateral hind paw. Nociceptive behaviors were expressed as the number of flinches and counted immediately within 5 min after injection (29, 35).

### Data analysis

All data were expressed as mean ± SEM and statistically compared using Student's *t* test or analysis of variance (ANOVA), followed by Bonferroni post hoc test. The nonlinear curve-fitting program ALLFIT was used for statistical analysis of the concentration–response data.

## Data availability

All data and analyses are included in the main text of the article.

## Ethics statement

The animal study was reviewed and approved by the Animal Research Ethics Committee of Hubei University of Science and Technology (2016-03-005).

## Conflict of interest

The authors declare that they have no conflicts of interest with the contents of this article.
